# Association between high and low ambient temperature and out-of-hospital cardiac arrest with cardiac etiology in Japan: a case-crossover study

**DOI:** 10.1186/s12199-017-0669-9

**Published:** 2017-07-13

**Authors:** Shin Yamazaki, Takehiro Michikawa

**Affiliations:** Environmental Epidemiology Section, Centre for Health and Environmental Risk Research, National Institute for Environmental Studies, Onogawa 16-2, Tsukuba, 305-8506 Japan

## Abstract

**Objective:**

The objective of the study was to examine the association between high and low temperature and out-of-hospital cardiac arrest (OHCA) with cardiac etiology.

**Methods:**

The study was conducted under a case-crossover design. Subjects were 97,500 patients aged 40 years or older with OHCA having a cardiac etiology in Tokyo, Osaka, and Fukuoka Prefecture from 2005 to 2012. We used national data with an Utstein-style resuscitation registration. Temperature was categorized into five categories with cut points of 5, 10, 24, and 30 °C. The reference category was 10–23.9 °C. Conditional logistic regression was used with adjustment for daily means of relative humidity, atmospheric pressure, and wind speed and daily amount of precipitation and hours of daylight.

**Results:**

Exposure to high temperature (≥30 °C) increased the risk of OHCA (OR = 1.11, 95% confidence interval (CI) 1.04–1.18). Further, low temperature (<5 °C) and relatively low temperature (5–9.9 °C) were also associated with OHCA (OR = 1.20, 95% CI 1.16–1.25; OR = 1.10, 95% CI 1.07–1.13, respectively). The temperature-OHCA association curves were U-shaped or J-shaped, and the association was more prominent among those aged 80 years or older.

**Conclusion:**

This study shows that the occurrence of OHCA with cardiac etiology is associated with low temperature. In addition, the occurrence is also associated with high temperature in those aged 80 years or older.

## Introduction

Many studies have provided estimates of death attributable to either heat or cold [1, 2]. For example, a multicountry study showed that mortality risk increased slowly for cold temperatures below the minimum mortality temperature and quickly at high temperature [3]. With regard to death caused by cardiovascular disease, a large American study found that 2-day mean extreme high and low temperatures were associated with increased risk of death [4]. In addition, a Chinese study showed that out-of-hospital coronary death (OHCD) from the Chinese death register was associated with 14-day mean temperature. The temperature-OHCD association curves in that study were U- or J-shaped and showed that extreme temperature significantly increased the risk of OHCD [5]. In contrast, a systematic review pointed out that the effects of temperature on cardiorespiratory morbidity appeared to be smaller and more variable than previous findings related to cardiorespiratory mortality [6]. In Japan, the occurrence of adult cases of out-of-hospital cardiac arrest (OHCA) with cardiac etiology increases with decreasing temperature during the day, in elderly people in particular [7].

Japan is presently aging more rapidly than any other country in the world. In 2014, the percentage of the population aged 65 and over (percentage of elderly) was 26.0% (previous year: 25.1%) and 12.5% for that aged 75 years old and over (the old elderly; 15.92 million people) [8]. With the progress of aging, it is expected that the number of people experiencing OHCA will increase.

A previous Japanese study suggested that the frequency of OHCA with cardiac etiology increases with decreasing temperature during the day [7]. However, analysis on the effect of extremely hot temperature on OHCA in that study was insufficient.

Here, we examined the association between extreme hot and cold temperatures and OHCA with cardiac etiology in Japan.

## Methods

### Study design

The study was conducted under a time-stratified case-crossover design, which is typically used to assess brief changes in risk associated with transient exposures [9, 10]. Case-crossover studies can be regarded as a special type of case-control study in which each case serves as its own control, thereby providing inherent control of potential confounding by fixed individual characteristics such as sex, race, diet, and age. “Time-stratified” indicates the method by which the control periods were chosen. Specifically, we stratified time into months to select days for control periods that fell on the same day of the week within the same month as the date of the occurrence of OHCA (day of the index period).

### Data collection

We used national data with an Utstein-style resuscitation registration. Subjects were patients with OHCA of presumed cardiac origin who were treated by emergency medical service personnel in Tokyo, Osaka, and Fukuoka Prefecture from January 1, 2005, to December 31, 2012. We restricted subject age to 40 years or older. The data were obtained from the Japanese Fire and Disaster Management Agency. Outcome was the occurrence of OHCA with cardiac etiology. Subject data included core data recommended in the Utstein-style reporting guidelines for cardiac arrest [11]. Cardiac arrest was defined as the cessation of cardiac mechanical activities and confirmed by the absence of sighs of circulation. The arrest was presumed to be cardiac origin unless it was caused by trauma, drowning, drug overdose, asphyxia, exsanguinations, or any other non-cardiac causes determined by physician in charge, in collaboration with the emergency medical service personnel.

Data on meteorological parameters, such as daily mean values for atmospheric pressure, relative humidity, temperature, wind speed, and total hours of daylight, were obtained from the Japan Meteorological Agency. We used a single meteorological observatory in each of Tokyo, Osaka, and Fukuoka. Temperature was categorized into five categories with the cut points of 5 °C (5th percentile), 10 °C (25th percentile), 24 °C (75th percentile) and 30 °C (95th percentile). The reference category was 10–23.9 °C.

### Statistical analysis

Conditional logistic regression was carried out using the PHREG procedures of SAS release 9.4 (SAS Institute, Inc, Cary, NC, USA). All tests were two-tailed, and alpha was set at 0.05. We computed ORs and their 95% CIs. Adjustment variables were daily means of humidity, atmospheric pressure, and wind speed and daily amount of precipitation and hours of daylight. We considered that air pollution could not be a confounder of ambient temperature-health effect association, because temperature is not affected by air pollution [12]. To examine the modified effects of age on the association between temperature and OHCA, data were grouped into the subject age groups of 40–59 years, 60–69 years, 70–79 years, 80–89 years, and 90 years or more.

The study protocol was approved by the ethical review board of the Japanese National Institute for Environmental Studies.

## Results

A total of 97,500 subjects with OHCA with cardiac etiology were identified. Table 1 shows that 68% were more than 70 years old and 58% were male. Table 1 also summarizes meteorological factors.

**Table 1:** Demographics of subjects and daily meteorological factors

		Tokyo		Osaka		Fukuoka	
Total, *n* (%)		50,698	(100)	34,283	(100)	12,519	(100)
Sex	Male	29,685	(59)	19,565	(57)	7145	(57)
	Female	21,013	(41)	14,718	(43)	5374	(42)
Age	40–59 years	6342	(13)	4146	(12)	1537	(12)
	60–69 years	8274	(16)	6016	(18)	2012	(16)
	70–79 years	12,935	(26)	9429	(28)	3234	(25)
	80–89 years	15,848	(31)	10,287	(30)	4061	(31)
	≥90 years	7299	(14)	4405	(13)	1675	(13)
Meteorological factors, mean (s.d.)							
	Temperature, °C	16.6	(8.0)	17.1	(8.4)	17.3	(8.0)
	Relative humidity, %	59.7	(15.4)	62.7	(10.7)	65.6	(11.5)
	Atmospheric pressure, hPa	1009.3	(6.7)	1005	(6.3)	1013.5	(6.8)
	Daily hours of daylight, hours	5.2	(4.0)	5.6	(3.9)	5.1	(4.0)
	Daily precipitation, mm	4.4	(12.7)	3.6	(9.6)	4.5	(13.3)
	Wind speed, m/s	3.1	(1.0)	2.4	(0.8)	2.8	(1.1)

**Fig. 1:**
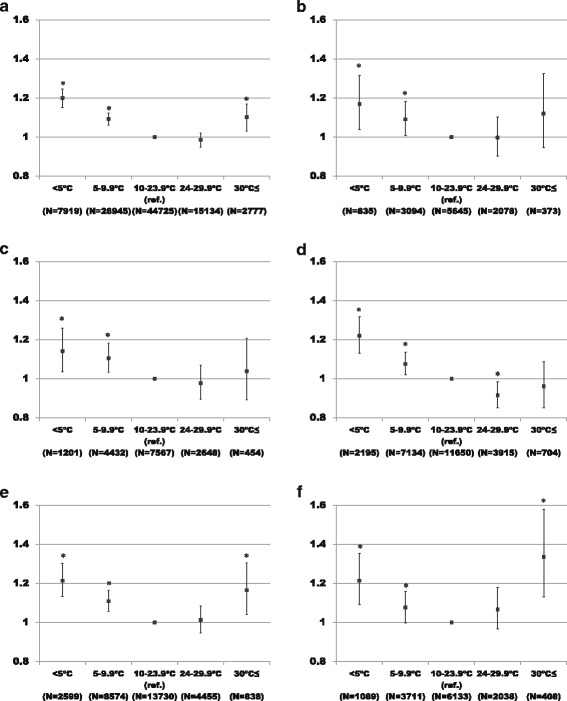
Odds ratio and 95% confidence intervals of the occurrence of out-of-hospital cardiac arrest with cardiac etiology by temperature. Adjustment variables were daily means of relative humidity, atmospheric pressure, and wind speed and daily amount of precipitation and hours of daylight. *P < 0.05. **a** Total. **b** Age 40–<60 years. **c** Age 60–<70 years. **d** Age 70–<80 years. **e** Age 80–<90 years. **f** Age ≥90 years

Figure 1 shows the association between temperature and OHCA with cardiac etiology. Compared to exposure to a mean temperature of 10–23.9 °C, exposure to a mean temperature of ≥30 °C was significantly associated with OHCA (OR = 1.11, 95% CI 1.04–1.18). We also observed that extremely low temperature (<5 °C) and relatively low temperature (5–9.9 °C) were associated with OHCA (OR = 1.20, 95%CI 1.16–1.25; OR = 1.10, 95% CI 1.07–1.13, respectively). The temperature-OHCA association curves were U- or J-shaped. The association was more prominent among those aged 80–89 years (Fig. 1e) and among those aged 90 years or older (Fig. 1f). The significant association between high temperature (≧30 °C) and OHCA was lost among subjects aged less than 80 years (Fig. 1b–d).

In addition to temperature, humidity was also associated with OHCA (RR = 0.99 for a 10% increase in relative humidity; 95% CI 0.98–1.00). None of the other meteorological factors was associated with OHCA.

## Discussion

These finding support an association between both high and low temperature and cardiovascular disease. We also noted that the association between high and low temperature and OHCA was particularly observed in those aged 80 years or older. Compared to the risk of OHCA in days of 10–23.9 °C, the risk increased about 20% in days of 30 °C or more in those aged 80 years or older. These findings have important implications for the planning of public health interventions to minimize the health consequences of adverse temperatures.

With regard to the susceptibility of elderly persons to temperature, previous studies have also shown that the association between extreme hot and cold temperatures and out-of-hospital coronary death is particularly high in higher aged groups [5].

A previous Japanese study with 28,806 OHCA patients suggested that low temperature was associated with OHCA with cardiac etiology, but high temperature was not [7]. One of the reasons of this difference between the results of the previous study and our study was the difference of sample size. Our study analyzed more number of patients than the previous study. In addition, our study was conducted using case-crossover analysis. The study design might be more appropriate than cross-sectional analysis which was used in the previous study.

Several limitations to the present study warrant mention. First, our study design was limited with respect to control of within-person confounding, which is still possible for multiple, correlated transient factors that change over time within a subject. Second, the estimated Odds ratios in this study may have suffered from non-differential misclassification, causing our results to be biased toward null, as single values for meteorological data were assigned to all individuals living in a certain area. Third, the association of OHCA with weather variables warrants more discrete analysis, such as the association between OHCA and temperature adjusted for air pollutants, OHCA and daily lowest temperature, OHCA and daily highest temperature, OHCA and daily variation of temperature, and time lag structure of the association between OHCA and temperature.

Further studies are needed to assess the association between hourly temperature and hourly onset of OHCA and the association between temperature and OHCA in higher and lower latitude regions and to confirm our findings.

In conclusion, this study shows that the occurrence of OHCA with cardiac etiology is associated with low temperature. In addition, the occurrence is also associated with high temperature in those aged 80 years or older. Informing local residents of temperature may help prevent OHCA events in that area.

## Abbreviations

OHCA: Out-of-hospital cardiac arrest
OHCD: Out-of-hospital coronary death
